# Development of a patient safety care activity scale for clinical nurses in Korea

**DOI:** 10.1186/s13690-021-00596-2

**Published:** 2021-05-17

**Authors:** Ya Ki Yang

**Affiliations:** Department of Nursing, College of Medicine, Wonkwang University, #460 Iksan-daero, Iksan City, Jeollabukdo 54538 South Korea

**Keywords:** Patient safety, Safety management, Activity, Nursing, Scale

## Abstract

**Background:**

The aim of this study was to develop a scale to measure patient safety care activities for clinical nurses and to verify validity and reliability it.

**Methods:**

Literature review and expert consultation were utilized to develop the scale of the Patient Safety Care Activities. The validity and reliability analyses were conducted with 428 nurses working at 5 general hospitals. Exploratory factor analysis of the scale was performed, and convergent and discriminant validity as well as internal consistency reliability were determined.

**Results:**

Eight subcategories (security, patient identification, operation (invasive procedure), medication, blood transfusion, management of infection, management of falls & sores, management of firefighting) with 44 items were validated to measure patient safety care activities. Convergent and discriminant validity indicated the applicability of the eight-factor Patient Safety Care Activities scale. The reliability of the Patient Safety Care Activities Scale was acceptable, with Cronbach’s a = .88 ~ .95.

**Conclusion:**

The developed scale showed content, construct validity, and reliability, as well as convergent validity for each item and discriminant validity between the factors. This makes it suitable for use in a diverse range of future studies on patient safety care activity.

## Background

Hospital safety incidents refer to all types of errors, mistakes, and accidents that occur in a hospital regardless of the harm caused on the patient [1]. Aging of population, increased prevalence of chronic diseases, advances in medical technology, and establishment of a national health insurance system improved people’s access to healthcare services, and as a result, unexpected safety incidents are bound to occur. Failure to promote patient safety in a healthcare facility threatens patients’ lives as well as undermining patients’ trust in healthcare professionals and facility and prolonging hospital stay, resulting in poor healthcare quality and financial loss [2]. Therefore, ensuring patient safety must be the most important responsibility of healthcare facilities, and nurses should demonstrate clear strategies and objectives to enhance patient safety while providing care [3].

The Institute of Medicine report that proposed patient safety incidents as a major culprit deteriorating the quality of healthcare service [4] sparked much interest in healthcare service errors in the field of medicine. South Korea conducts healthcare facility accreditation evaluations, which includes items about patient safety, every 3 years since 2004, and with an implementation of stricter evaluation criteria pertaining to patient safety from 2007, the evaluation now includes more specific items such as marking of surgical site and patient identification before medication administration and sampling for diagnostic purposes [5]. Recently, there have been more effort to institutionalize patient safety, such as increased acquiring of or effort to acquire an accreditation from the Joint Commission International (JCI) among tertiary hospitals.

Safety care activities are the identification, improvement, and prevention of problems or potential problems that may arise during the process of treatment and care [6]. The Joint Commission (TJC) standard is the gold standard in patient safety management in hospital practice, and it consists of items such as accuracy of treatment, efficiency of communication among healthcare professionals, safety of drug use (accurate and safe administration), and reduction of risk of injury from falls. Nursing work is closely related to patient safety in types of work such as infection control, medication administration, fall management, and facility management, and because nurses are the final healthcare providers who can detect and address errors pertinent to medication and treatment [7], nurses’ interests and perception of safety care activities are critical factors in improving patient safety.

Nurses, who directly deliver healthcare service to patients, are responsible for a broad scope of work, and safety care activities encompass a variety of areas. Thus, standardized guidelines for and an instrument to assess safety care activities would contribute to enhancing the quality of safety care activities and patient safety outcomes through accurate and consistent management and assessment. However, the currently available instruments to measure safety care activities only contain some of the areas in the healthcare accreditation standard, such as only focusing on infection, communication, falls, medical equipment management, surgery/invasive procedures, staff safety, or fire safety [8], or only partially cover a variety of areas, including falls, participant training, infection, facility inspection, fire, patient identification, communication, medication administration, and blood transfusion [9]. The objective of to directly compare safety care activities is to prepare guidelines for integrated safety management activities. Existing tools were intended to measure falls, bedsores, and fire prevention in part, and most available tools are non-standardized tools with unestablished reliability and validity. Furthermore, the validated tools for medication administration safety [10] and falls and bedsore care [11–13] only assess a single safety care activity. As a result, these tools are significant for patient safety in the corresponding areas but cannot present the level of comprehensive patient safety activities. In addition, studies also measure the awareness of patient safety culture to indirectly propose the level of safety care activities with an argument that individuals with a high awareness of patient safety culture will engage in a high level of safety care activities [8, 9, 14].

Thus, this study aims to develop and validate an instrument that accurately measures the level of safety care activities of clinical nurses to provide foundational data for continuous improvement of safety care activities. This study aims to develop and validate an instrument that comprehensively measures safety care activities in order to promote a patient safety culture and safety care activities. The specific objectives are as follows:

- Develop an instrument to measure safety care activities of clinical nurses.
- Test the validity and reliability of the developed Safety Care Activity Scale.

## Methods

### Research design

This is a methodological study for developing and validating the Safety Care Activity Scale for clinical nurses. The development of the scale was carried out in the steps of preliminary item composition, pre-test, and validity, reliability verification. This study was divided into the scale development stage (literature review, expert review, pre-test) and scale evaluation stage (questionnaire research).

### Scale development process

The Safety Care Activity Scale was developed based on the scale development guidelines by DeVellis [15].

#### Step 1: preliminary item composition

To compose the items for the scale, key concepts and components of safety care activities were examined based on the healthcare organization accreditation criteria in Korea and literature review. At the stage of literature reviewing, using literature search programs such as the cumulative index to nursing and allied health literature, Pubmed, and the Korea Academic Information Institute. The main conceptual words of this study were patient safety, safety management, activity, and instrument. The subcategories and items were compared among existing literature related to safety care activities. And focus group interview was conducted on seven nurses with at least 2 years of experience as nursing staff in the department dedicated to patient safety in a hospital. Semi-structured and open-ended questioning method was used in interview, and asked, ‘Please tell me about the nursing care necessary for patient safety.’ The time required for discussion was about 3 h. Interviews proceeded from each subject to the point of saturation of data that they thought were no longer coming out of new statements related to patient safety nursing activities. Participants were allowed to speak freely without being restricted, but all subjects were asked to speak at least once in a single question so that all subjects could participate and talk without being biased against only one subject. Analysis was carried out in 4 steps. In the first step, after transcripting the interview contents, the data were repeatedly read and a meaningful statement was drawn. In the second step, phrases or sentences with meaningful content related to patient safety nursing were selected and re-statemented in a general form that could represent them. In the third step, meaning was derived from meaningful statements and restatements as a step of constructing meaning. In step 4, the composed meanings were integrated and categorized into 12 subjects.

With reference to the criterion to use at least two times more items than the anticipated number in the preliminary scale [16], a total of 80 items were chosen. A total of 12 subcategories were chosen: security, patient identification, communication, surgery/invasive procedure, fall, infection, pressure injury, blood transfusion, medication administration, fire safety, facility and medical equipment management, and patient safety reporting system. Two nursing professors reviewed the validity of the preliminary items and revised it by a Korean language literary scholar.

#### Step 2: validity of preliminary item and pre-test

The validity of the preliminary items for the Safety Care Activity Scale was rated on a 4-point scale, with “highly relevant” (4), “quite relevant” (3), “somewhat relevant” (2), and “not relevant” (1). An expert panel of 10 experts, namely three nursing professors and seven nurses with at least 5 years of experience as nursing staff specializing in patient safety, was selected for the validity judgment [17]. The content validity of the 80 preliminary items of the Safety Care Activity Scale was assessed using item content validity index (I-CVI) and scale content validity index (S-CVI).

The I-CVI asks about the relevance, redundancy, and clarity of each item, and all items with an I-CVI for relevance of below .80 were deleted. The remaining items were assessed for redundancy (I-CVI > .80), and those rated to be redundant were deleted. Items rated to be unclear were revised, and a total of 55 items in 12 subcategories were identified. The S-CVI, which refers to the percentage of items rated as 3 or 4 by 10 experts, was .83.

A pre-test was conducted to examine the comprehensibility and reliability of the validated preliminary items as well as the time required to complete the survey. The pre-test was conducted on 30 staff nurses at W hospital from December 5, 2019 to December 8, 2019. None of items were not answered due to difficulty of understanding. The time required for the survey was about 20 min. The Cronbach’s α of the preliminary scale was .99, and none of the items increased the Cronbach’s α by .10 or higher when deleted. Further, the correlation between the item and all remaining items ranged from .34–.92, based on which all 55 items were selected to be included in the finalized scale.

#### Step 3: safety care activity scale verification (validity and reliability)

### Participants

Based on Gorsuch’s suggestion that the sample size for factor analysis should be at least 5–10 times larger than the number of items [18], 450 clinical nurses were selected in consideration of potential withdrawals. Nurses working in the insurance review division, health examination center, and outpatient setting without direct contact with patients were excluded.

### Data collection

A total of 441 questionnaires were retrieved (98% retrieval rate), and after excluding questionnaires with careless responses, 428 were included in the analysis. It took about 10–15 min to complete the survey, and data were collected from clinical nurses of 5 general hospitals in J province and G, S, P cities. The questionnaires were distributed and collected by the head of the nursing units in each hospital from January 6, 2020 to January 31, 2020. The heads of each nursing unit were informed about the purpose of the study, and data were collected from only those hospitals that provided an informed consent to participate in the study. The questionnaire contained the researchers’ contact information and email address so that the participants can direct all of their inquiries about study participation or questionnaire content to the researchers, and to ensure voluntary study participation, the written consent form contained an item about voluntary participation so as to allow nurses to autonomously determine their participation.

### Statistical analysis

The collected data were analyzed using the SPSS Statistics 24 program. The participants’ general characteristics were examined using descriptive statistics. The content validity of the preliminary items were calculated by CVI. Only the items with a CVI of .80 or higher [17] were selected and the opinions of experts were reflected in the item revision. In the course of item analysis, the correlation coefficient between each item and total items was calculated, and the criterion >.30 [19] was applied. The verification of the construct validity of the preliminary scale was calculated by exploratory factor analysis. Kaiser-Meyer-Olkin (KMO) values were calculated and Bartlett’s sphericity was verified to determine the possibility of exploratory factor analysis for verification of construct validity. As an exploratory factor analysis method, the main axis factor extraction method and the Verimax rotation method were used, and the factors were extracted based on the eigen value of 1.00 or higher, and the factor load was applied to the standard >.40 [19]. The criterion validity was analyzed using Pearson’s correlation coefficients with reference to the Korean modified version of the Hospital Survey on Patient Safety Culture, originally developed by the US Agency for Healthcare Research and Quality [20] and translated and modified into Korean by Kim et al. [21], which is the most widely used instrument to measure safety care activities in Korea. The reliability of the scale was tested based on Cronbach’s α for internal consistency.

### Ethical considerations

The author informed the participants of the purpose, procedure, and confidentiality of the study to the participants prior to data collection, and an informed consent was obtained from the volunteers. The consent form specified that all personal information obtained will only be used for research purposes and that participants have the freedom to withdraw from the study at any time. This study was approved by the institutional review board (IRB) at W University (IRB No: WKIRB-202002-SB-004) before data collection.

## Results

### Participant characteristics

The mean age of the participants was 30.42 years, and 98.1% were female. Highest education was bachelor’s degree (73.4%), associate degree (22.9%), and masters or higher (3.7%). The mean total clinical career was 7.91 years, and 30.8% worked in internal medicine ward. There were more healthcare organizations that have been accredited (93.0%) than those that have not been accredited (7.0%). The majority (93.5%) of the participants had prior training related to safety care activities, and 99.5% stated that their hospital has a patient safety division (Table 1).

**Table 1:** Safety care activities to the general characteristics of participants (*n* = 428)

Characteristics	Categories	N (%)
Sex	Male	8 (1.9)
	Female	420 (98.1)
Age (year)	≤25	142 (33.2)
	26 ∼ 30	142 (33.2)
	31 ∼ 35	52 (12.1)
	36 ∼ 40	28 (6.5)
	≥41	64 (15.0)
Education level	3-year college	98 (22.9)
	Bachelor	314 (73.4)
	≥Master	16 (3.7)
Total career (year)	<4	196 (45.8)
	4 ∼ 7	74 (17.3)
	8 ∼ 14	72 (16.8)
	≥15	86 (20.1)
Career present unit (year)	<2	160 (37.4)
	2 ∼ 3	134 (31.3)
	4 ∼ 6	82 (19.2)
	≥7	52 (12.1)
Patient safety education	Yes	400 (93.5)
	No	28 (6.5)
Experiences on accreditation	Yes	398 (93.0)
	No	30 (7.0)
Type of unit	Internal medicine ward	132 (30.8)
	Surgical ward	96 (22.4)
	Emergency room	54 (12.7)
	Intensive care unit	78 (18.2)
	Operation room/Delivery room	68 (15.9)
QI department	Yes	426 (99.5)
	No	2 (0.5)

### Validity

#### Item analysis

The mean score of each item was 2.47–3.89, with a standard deviation of 0.62–1.06. The absolute value of skewness was 0.01–0.59, and that of kurtosis was 0.01–1.06, which was below 2 and thus was deemed acceptable. The correlation coefficient for each item with the total score was .45–.71, meeting the cutoff of .30 or higher. All items were suitable (Table 2).

**Table 2:** Item Analysis (*n* = 428)

Item No	M	SD	Skewness	Kurtosis	Item-total correlation
1	3.36	0.56	−0.15	− 0.76	.417
2	3.33	0.59	− 0.25	− 0.64	.564
3	3.27	0.70	− 0.67	0.15	.565
4	3.40	0.60	− 0.57	0.12	.491
5	3.51	0.50	−0.06	− 1.01	.561
6	3.43	0.51	0.06	−1.51	.614
7	3.38	0.51	0.26	− 1.41	.679
8	3.68	0.47	−0.76	− 1.43	.640
9	3.72	0.45	−0.98	−1.04	.582
10	3.67	0.47	−0.74	−1.46	.634
11	3.67	0.49	−0.98	−0.46	.627
12	3.47	0.52	−0.07	−1.52	.674
13	3.23	0.76	−0.87	0.55	.624
14	3.41	0.54	−0.08	−1.07	.706
15	3.57	0.49	−0.30	−1.92	.738
16	3.56	0.58	−1.21	1.94	.663
17	3.58	0.53	−0.70	−0.74	.736
18	3.49	0.58	−0.71	0.42	.640
19	3.48	0.55	−0.59	0.30	.739
20	3.54	0.54	−0.54	−0.95	.713
21	3.37	0.68	−0.89	0.72	.663
22	3.46	0.53	−0.14	−1.34	.730
23	3.63	0.49	−0.64	−1.30	.789
24	3.48	0.52	−0.11	−1.52	.777
25	3.57	0.55	−0.77	−0.49	.638
26	3.67	0.48	−0.84	−0.96	.729
27	3.67	0.48	−0.87	−0.92	.715
28	3.66	0.48	−0.82	−1.01	.742
29	3.68	0.48	−0.92	−0.82	.702
30	3.67	0.49	−0.95	−0.52	.653
31	3.66	0.49	−0.93	−0.57	.631
32	3.58	0.51	−0.55	−1.21	.706
33	3.62	0.50	−0.62	−1.33	.716
34	3.56	0.52	−0.45	−1.32	.692
35	3.59	0.50	−0.47	−1.50	.745
36	3.60	0.51	−0.64	−1.09	.790
37	3.58	0.54	−0.79	−0.51	.731
38	3.58	0.55	−1.03	1.03	.736
39	3.49	0.63	−1.28	2.59	.731
40	3.55	0.55	−1.05	2.01	.727
41	3.54	0.54	−0.70	0.46	.740
42	3.51	0.55	−0.65	0.37	.731
43	3.57	0.54	−0.73	− 0.61	.689
44	3.59	0.49	−0.36	−1.88	.764
45	3.53	0.54	−0.57	−0.83	.693
46	3.53	0.51	−0.24	−1.68	.709
47	3.23	0.60	−0.14	−0.49	.561
48	3.27	0.58	−0.11	−0.50	.570
49	3.14	0.66	−0.16	−0.72	.568
50	3.20	0.61	−0.14	−0.49	.565
51	3.48	0.52	−0.11	−1.52	.742
52	3.54	0.50	−0.17	− 1.98	.762
53	3.49	0.50	0.06	−1.01	.777
54	3.43	0.52	−0.03	−1.34	.671
55	3.46	0.52	−0.04	−1.52	.716

*SD* Standard deviation

#### Exploratory factor analysis (EFA)

EFA was performed to test the construct validity of the tool. Prior to the EFA, Kaiser-Meyer-Olkin (KMO) and Bartlett test of sphericity were performed to determine the suitability of the data for factor analysis. The KMO value was .93, and Bartlett test of sphericity also confirmed that the data has common factors and is suitable for EFA (*p* < .001).

EFA was performed with varimax rotation to test the construct validity of 55 items. Eight factors had an eigenvalue of 1.0 or higher, with a cumulative total variance explained of 76.0%. While 12 factors theoretically established, only eight were identified. Pressure injury management and fall management emerged as a single factor, and factor analysis was performed again after removing 6 items also loaded under another factor and 5 items with a factor loading of .40 or below. After removing 11 items and conducting factor analysis on 44 items, the KMO value was .93, and Bartlett test of sphericity led to a χ^2^ statistic of 21,141.70 (*p* < .001). Eight factors had an eigenvalue for 44 items of 1.0 or higher, and cumulative variance explained was 76.91% (Table 3).

**Table 3:** Exploratory factor analysis (Final Stage) (*n* = 428)

Item No.	Communality	Factors							
		1	2	3	4	5	6	7	8
1	.508	.048	.255	**.567**	−.122	−.020	.092	.118	.286
2	.744	.122	.011	**.777**	.193	.117	.012	.073	.263
3	.629	.045	.027	**.666**	.297	.187	.221	.046	.092
4	.607	.088	.122	**.707**	.029	.150	.240	−.052	.029
5	.707	.201	.038	**.754**	.102	.149	.225	.094	−.070
6	.740	.213	.149	**.777**	.091	.123	.091	.195	.005
7	.757	.226	.032	**.710**	.097	.262	.172	.253	.171
8	.831	.197	.155	.238	.120	.081	**.785**	.199	.187
9	.871	.202	.189	.239	.122	.013	**.845**	.0410	.084
10	.882	.119	.165	.317	.159	.077	**.806**	.0990	.224
11	.624	.154	.409	.245	.190	.073	**.552**	.0450	.155
16	.829	.364	.113	.152	.094	.152	.285	.120	**.730**
17	.822	.306	.194	.242	.118	.160	.328	.210	**.664**
18	.679	.272	.153	.196	.256	.247	.171	−.018	**.622**
20	.814	.091	.400	.289	.206	.121	.077	**.602**	.370
21	.759	.07	.212	.244	.211	.207	.100	**.599**	.439
22	.762	.329	.202	.096	.335	.179	.223	**.633**	.094
23	.793	.354	.509	.215	.168	.153	.189	**.518**	.088
25	.738	.424	.221	.163	.313	−.014	.034	**.613**	−.088
26	.768	.432	**.582**	0.15	.169	−.007	.250	.356	.046
27	.765	.276	**.644**	.114	.311	−.028	.248	.280	.155
28	.833	.224	**.712**	.136	.356	.115	.298	.098	.136
29	.790	.303	**.700**	.122	.333	.056	.266	.029	.089
30	.821	.228	**.810**	.081	.241	.108	.057	.133	.125
31	.789	.289	**.787**	.057	.217	.073	.050	.156	.053
32	.787	.216	.392	.134	**.707**	.132	.091	.161	.132
33	.861	.243	.313	.150	**.794**	.116	.121	.123	.093
34	.844	.265	.197	.187	**.797**	.045	.164	.184	.045
35	.761	.295	.324	.031	**.619**	.187	.192	.267	.203
36	.818	.370	.468	.068	**.570**	.144	.190	.175	.215
37	.708	.362	.334	.143	**.601**	.111	.096	.225	.106
38	.666	**.588**	.278	.195	.266	.106	.028	.207	.282
39	.783	**.722**	.213	.165	.061	.142	.102	.165	.357
40	.750	**.743**	.203	.092	.207	.117	.203	.099	.204
41	.786	**.768**	.173	.150	.257	.120	.182	.089	.146
42	.779	**.745**	.143	.161	.281	.197	.073	.022	.232
43	.758	**.687**	.387	.088	−.044	.132	.164	.260	.122
44	.747	**.642**	.406	.152	.196	.163	.167	.232	.020
45	.693	**.621**	.121	.280	.397	.234	.003	.044	−.013
46	.768	**.778**	.213	.108	.220	.175	.133	.091	.018
47	.912	.212	.113	.170	.076	**.901**	.090	.014	.022
48	.881	.294	.083	.117	.096	**.864**	.064	−.028	.109
49	.835	.128	.029	.275	.162	**.823**	−.028	.160	.108
50	.865	.091	.063	.197	.072	**.859**	.074	.174	.186
Eigen value		20.71	3.70	2.72	1.94	1.74	1.47	1.31	1.03
Explained variance (%)		15.31	12.02	10.75	10.36	8.38	7.64	6.62	5.83
Cumulative explained variance (%)		15.31	27.34	38.08	48.44	56.82	64.46	71.08	76.91

KMO = .93, Bartlett’s test: χ^2^ = 21,141.70 (*p* < .001)

Factor analysis generated 44 items under 8 factors, and among these factors, those that contain the identical items as that in the preliminary scale development was given the same name as that used in the development stage. Management of falls and management of sores were identified to be a single factor, and thus was named management of falls and sores. Factor analysis confirmed 7 items for security, 4 items for patient identification, 3 items for operation (invasive procedure), 5 items for medication, 6 items for blood transfusion, 6 items for management of infection, 9 items for management of falls and sores, and 4 items for management of firefighting.

#### Convergent validity and discriminant validity

To test the convergent validity and discriminant validity of each item, multi trait-multi item matrix analysis was performed. The correlation between each item with their overarching factor met the cutoff of .40 or higher with a range of .77–.94, and so the convergent validity of each item was established. Discriminant validity is deemed established when the difference in the correlation coefficient for the item with its overarching factor and the correlation coefficient for the item with another factor is more than twofold higher than the standard error of the correlation coefficient, and the results confirmed that none of the items markedly deviated from the criterion. Thus, discriminant validity was established (Table 4).

**Table 4:** Multi-trait/multi-item matrix for item convergent and item discriminant (*n* = 428)

Factor	Item No	Correlation between each item and total scores of sub-factor								2*SE
		F1	F2	F3	F4	F5	F6	F7	F8	
Security	1	**.632**	.360	.344	.349	.300	.222	.276	.185	.315
	2	**.818**	.389	.457	.450	.279	.343	.386	.360	.149
	3	**.785**	.475	.390	.424	.285	.406	.346	.368	.133
	4	**.768**	.425	.341	.308	.268	.257	.304	.319	.133
	5	**.787**	.469	.369	.417	.316	.318	.392	.362	.091
	6	**.819**	.432	.381	.500	.377	.396	.441	.361	.134
	7	**.836**	.502	.496	.560	.344	.421	.494	.492	.143
Patient identification	8	.502	**.898**	.566	.499	.505	.454	.480	.266	.088
	9	.464	**.924**	.475	.398	.484	.431	.443	.191	.082
	10	.560	**.929**	.561	.477	.492	.465	.436	.273	.104
	11	.460	**.800**	.489	.475	.596	.503	.475	.249	.062
Operation (Invasive procedure)	16	.425	.533	**.929**	.514	.457	.452	.593	.379	.104
	17	.520	.604	**.928**	.611	.537	.518	.599	.401	.114
	18	.435	.462	**.846**	.452	.443	.497	.542	.429	.076
Medication	20	.503	.462	.565	**.855**	.633	.576	.509	.343	.123
	21	.477	.430	.553	**.819**	.493	.532	.472	.394	.123
	22	.395	.468	.490	**.850**	.595	.648	.609	.373	.077
	23	.527	.463	.433	**.811**	.636	.676	.697	.369	.081
	25	.348	.328	.331	**.776**	.562	.602	.592	.219	.049
Blood transfusion	26	.398	.537	.495	**.687**	.847	.635	.680	.236	.078
	27	.362	.546	.505	.683	**.885**	.696	.602	.207	.071
	28	.392	.609	.510	.632	**.906**	.720	.604	.316	.079
	29	.364	.553	.468	.565	**.883**	.690	.621	.258	.082
	30	.294	.434	.422	.579	**.876**	.640	.569	.269	.059
	31	.268	.409	.387	.558	**.858**	.626	.585	.234	.060
Management of infection	32	.381	.435	.448	.636	.675	**.885**	.583	.335	.056
	33	.389	.439	.464	.619	.664	**.907**	.587	.331	.043
	34	.406	.453	.428	.628	.603	**.881**	.574	.274	.027
	35	.359	.479	.521	.684	.674	**.870**	.641	.376	.055
	36	.381	.527	.557	.682	.774	**.910**	.708	.369	.082
	37	.393	.450	.466	.650	.662	**.863**	.652	.346	.075
Management of falls & sores	38	.422	.422	.582	.617	.615	.639	**.793**	.374	.070
	39	.424	.445	.628	.595	.539	.528	**.861**	.390	.096
	40	.369	.476	.563	.557	.577	.599	**.854**	.360	.082
	41	.406	.471	.545	.590	.584	.613	**.873**	.373	.068
	42	.404	.398	.579	.548	.537	.603	**.860**	.434	.060
	43	.341	.455	.515	.581	.629	.493	**.813**	.343	.061
	44	.424	.477	.487	.647	.698	.652	**.830**	.383	.083
	45	.448	.343	.427	.530	.497	.601	**.766**	.446	.061
	46	.358	.400	.469	.547	.572	.583	**.860**	.379	.051
Management of fire fighting	47	.397	.268	.384	.340	.284	.345	.436	**.943**	.042
	48	.361	.251	.441	.326	.283	.365	.489	**.922**	.035
	49	.462	.227	.386	.435	.250	.364	.407	**.910**	.074
	50	.427	.280	.446	.426	.254	.336	.386	**.921**	.084

*S.E* Standard Error

#### Criterion validity

With reference to the study findings that safety care activities increase with increasing awareness of patient safety culture [8–10, 12], the criterion validity was tested with reference to a patient safety culture scale. The Hospital Survey on Patient Safety Culture developed by AHRQ [19] and translated and modified into Korean by Kim et al. [21] was used, and the scale consists of 44 items in 6 subscales, including 18 items for hospital work environment, 4 items about supervisor/manager’s attitude, 6 items about communication, 3 items for frequency of events reported, 1 item for level of general patient safety, 11 items for hospital climate, and 1 item for reported number of incidents. With the exception of the 1 item for reported number of incidents, all items are rated on a 5-point Likert scale. To prevent response bias, negatively worded items were included, which were reverse coded for analysis. A higher score indicates greater awareness of patient safety culture. The Cronbach’s α of the scale was .78 and the reliability of each subscale ranged from .67–.84 in the study by Kim et al. [21]. In this study, the reliability of the entire scale was .84, and that of each subscale ranged from .62–.87.

There was a statistically significant correlation between awareness of patient safety culture and safety care activities (*r* = .51, *p* < .001), and the correlations between patient safety culture and all subscales of safety care activities were also significant (Table 5).

**Table 5:** Correlation between patient safety culture and patient safety nursing activity instrument (*n* = 428)

		Patient Safety Culture						
		Total	①	②	③	④	➄	⑥
		r (*p*)						
Patient Safety Nursing Activity	Total	.51^**^	.48^**^	.43^**^	.44^**^	.20^**^	−.30^**^	.22^**^
	⑦	.49^**^	.43^**^	.41^**^	.50^**^	.13^**^	−.35^**^	.38^**^
	⑧	.39^**^	.33^**^	.36^**^	.40^**^	.13^**^	−.28^**^	.28^**^
	⑨	.43^**^	.40^**^	.37^**^	.43^**^	.14^**^	−.30^**^	.30^**^
	⑩	.44^**^	.78^**^	.46^**^	.36	.41^**^	−.31^**^	.31^**^
	⑪	.43^**^	.81^**^	.43^**^	.38	.50^**^	−.37^**^	.39^**^
	⑫	.44^**^	.39^**^	.35^**^	.40	.21^**^	−.28^**^	.31^**^
	⑬	.45^**^	.39^**^	.36^**^	.39	.22^**^	−.28^**^	.31^**^
	⑭	.48^**^	.46^**^	.43^**^	.37	.33^**^	−.35^**^	.34^**^

① Hospital work environment ② Supervisor/Manager’s attitude ③ Communication ④ Frequency of events reported ⑤ Level of general patient safety ⑥ Hospital climate ⑦ Security ⑧ Patient identification ⑨ Operation (Invasive procedure) ⑩ Medication ⑪ Blood transfusion ⑫ Management of infection ⑬ Management falls & sores ⑭ Management of fire fighting

** *p* < .001

### Reliability

Internal consistency, as measured with Cronbach’s α, was .96, and the Cronbach’s α for each subcategory was as follows: security .89, patient identification .91, operation (invasive procedure) .88, medication .90, blood transfusion .94, management of infection .95, management of falls and sores .95, and management of firefighting .94. The cutoff for internal consistency measured with Cronbach’s α is .70 or higher for a new instrument and .80 or higher for an existing instrument. None of the items increased the Cronbach’s α by .10 or higher when deleted. The coefficient was .80 for the entire items and .70 or higher for all of the subscales, thereby verifying reliability.

### Finalized safety care activity scale

After empirically establishing the validity of the scale via EFA, the Safety Care Activity Scale was finalized to 44 items in 8 subcategories. The rating scale was a 4-point Likert scale from 1 “strongly disagree,” 2 “disagree,” 3 “agree,” and 4 “strongly agree,” where a higher score indicates greater compliance with safety care activities. The finalized Safety Care Activity Scale consisted of 7 items for security, 4 items for patient identification, 3 items for operation (invasive procedure), 5 items for medication, 6 items for blood transfusion, 6 items for management of infection, 9 items for management of falls and sores, and 4 items for management of firefighting (Appendix).

## Discussion

This study was designed to develop an instrument to measure safety care activities of clinical nurses and attempted to present foundational data for improving nursing practice related to patient safety. A standardized patient safety-related nursing activities were identified via a literature review, and the preliminary items for safety care activities were written based on the experiences of healthcare organization accreditation evaluation and current experiences with safety care activities through a focus group interview with 7 clinical nurses who are in charge of patient safety in a general hospital.

The content validity of the preliminary items was tested, and the results showed an I-CVI of .88 and S-CVI of .83, indicating high validity. The validity was tested in three stages of appropriateness, redundancy, and clarity. The comprehensibility and reliability of the preliminary items and time required to complete the survey were examined through a pilot survey, based on which the preliminary Safety Care Activity Scale was developed. To validate the preliminary scale, the reliability and validity of the scale were tested using a sample consisting of randomly selected clinical nurses from three university hospitals across regions. In terms of the criterion validity, patient safety culture was strongly correlated with safety care activities, and this not only establishes the criterion validity of the developed Safety Care Activity Scale but also supports important previous findings that safety care activity increases with increasing awareness of patient safety culture [8, 9, 14], which adds to the significance of this study.

The reliability of the Safety Care Activity Scale developed in this study as measured with Cronbach’s α was .96. Although this cannot be directly compared with previous finding due to the differences in the subscales and items, it is still higher than the Cronbach’s α found in other studies that used a safety care activity tool (.92–.95) and is above the criterion of .90 for high reliability for a socio-psychological instrument. Hence, the scale developed in this study can be deemed to have a high reliability.

The finalized Safety Care Activity Scale consisted of 44 items in 8 subcategories (security, patient identification, operation (invasive procedure), medication, blood transfusion, management of infection, management of falls and sores, and management of firefighting. A 4-point Likert scale was chosen because using a 3-point or 5-point rating scale may lead to problems related to a neutral category [17]. The finalized tool consisted of a quite large number of items (44), as the study was conducted on nurses in general hospitals that contain a number of units (e.g., medical, surgical, OR, ER, ICU) and not on nurses of a specific unit.

This study proposed a novel factor known as security that is not included in the existing safety care activities. Personal medical information can be defined as all records about patients obtained through treatment. Due to the recent development of network-based medical devices, sensitive personal information including patient information is being collected, distributed, and utilized through the network. Above all, the need for medical security is even greater in that medical information is directly connected to life and body. However, recognition of the importance of domestic medical security is not high, and specific protection measures are insufficient. In addition, most of the workers in medical institutions have low interest in medical information and are not aware of the importance. Since various institutions, including insurance companies as well as medical institutions, share patient personal information, negligence in managing the patient’s medical information can lead to secondary damage through a combination of the patient’s personal financial records and biometric information. Even though they realize the importance of medical security and try to improve the security of personal information, in most cases they do not know how to practice concretely. Therefore, education on prevention of security accidents and education on countermeasures is necessary through online and offline education. It is an opinion that it is not just time-filling education, but case-centered concrete practice plan education. Security is an essential area to be included in safety care activities in that it allows the prediction of nurses’ activities to protect patients’ medical information in obligatory environments such as hospitals and help establish policies or guidelines to promote the protection of medical information.

Patient identification is at the basis of all nursing activities, and it included contents about the requirement to identify a patient with two or more pieces of information and the need to implement a rule and regulation to require two healthcare professionals to identify a patient before blood transfusion. This not only satisfies one of the six goals proposed by JCI, which states that comprehensive effort should be made to develop measures and protocol to identify patients correctly using two identifiers [22] and method and timing of patient identification suggested by the Korea Institute for Healthcare Accreditation (KOIHA) [23] but also presents the details related to patient identification before blood transfusion, sampling for a test, and giving treatment, rendering the tool as more comprehensive.

Operation (invasive procedure) management had a relatively low impact on the overall safety care activities, but this may be attributable to the fact that many of the nurses were stationed in nonsurgical units. The subscale includes items about patient involvement in marking of surgical site and checklist for verification before and immediately before surgery (invasive procedure).

In Korea, medication errors account for the vast majority of errors in therapeutic nursing activities directly delivered to patients [10]. Approximately 56.2% of medication errors are made by a nurse [24], and 79% of these cases were found to be caused by “carelessness,” where the error could have been prevented with more precaution [25]. The tool developed in this study not only includes the JCI guidelines about labeling for safe medication administration and developing a list for each patient [22] and the “label high-risk and high-alert medications for storage” in the category “accurate communication among healthcare professionals” proposed by the KOIHA [23] but also classifies the items by specific area, which would help actively prevent errors.

Blood transfusion is closely linked to nurses’ roles of patient identification, blood type verification, infusion of correct blood, and monitoring of adverse reactions, and blood transfusion errors can be prevented with nurses’ safety management activities [26]. In Korea, the Korean Society of Blood Transfusion developed the guidelines for blood management and blood transfusion in 2002, and healthcare organization accreditation evaluation includes meticulous entry of patient’s blood sample information, appropriateness of testing before transfusion, time from dispensing of blood to transfusion, and monitoring of adverse reactions during transfusion in the evaluation of appropriateness of blood transfusion management [23]. The present tool contains contents about monitoring of adverse reactions during blood transfusion and additionally includes measures to be taken upon onset of adverse reactions.

In this study, nurses were found to perceive infection control as the most important. This may be attributable to the increased perception among healthcare providers that infection control is the only way to ensure safety for themselves and patients, owing to the continual exposure to novel infections such as MERS and COVID-19 in recent years. Infection control is also one of the 13 domains and a significant part of the KOIHA [24] evaluation criteria, so this study is significant in that it developed a tool that can be applied to clinical nurses of all areas.

Management of falls and sores has emerged as a serious patient safety-related issue in healthcare organizations [13, 25]. Falls and pressure injuries are predictable and preventable health problems, as opposed to an unavoidable accident, whose responsibility often falls on nurses [13, 27], and so nurses must pay close attention during the duration of the patient’s hospital stay. Preventing, as opposed to treating after the fact, falls and pressure injuries is helpful for both patients and caregivers and can contribute to improving the quality of care. The present tool emphasized the importance of prevention by including a more detailed description of fall and pressure injury prevention.

Healthcare facilities are at a high risk for fire due to the numerous small wards with long aisles and built-in combustible materials, such as beddings, medicines, and medical equipment. Moreover, the persons occupying the facilities consist of unspecified users and patients with reduced mobility, which may lead to more serious damages and casualties in a natural or man-caused fire compared to other types of buildings. Nurses must be aware of the shelters within the facility and patients’ capability of evacuation in advance such that they can guide people to safely evacuate from the building in case of a fire.

The scale developed in this study can be the basis for providing better nursing care by raising concern and awareness about patient safety to the nurses who care for the patient in the nearest place, and further to protect the nurses themselves. If clinical nurses working in hospitals use this scale, they will be able to perform safe nursing by identifying problems related to patient safety nursing activities and improvement directions more conveniently. In addition to the subcategories included in this scale, a review of existing revealed several other areas related to safety care activities, including transport care, suicide, crisis management, and emergency responses.

In this study, areas that are only applicable to specific units or subset of patients were excluded, so these areas should be added as needed in subsequent studies. In addition, there is a limitation that the reliability of the scale was verified only by the internal consistency of the item. Therefore, it is necessary to check the reliability of test-retest whether it is stably measured even if the size of the subject and the hospital are different as the final selected item. The tool developed in this study should be further modified and complemented to be applicable to nurses of various areas who are in charge of patient safety as the awareness of patient safety increases and improves. Further, subsequent studies may also examine the association with patient safety outcomes, such as the rate of safety incidents.

## Conclusions

In this study, a preliminary scale was developed based on a literature review and expert opinions about safety care activities, and the preliminary scale was validated on nurses of university hospitals to finalize the Safety Care Activity Scale. Use of this scale by clinical nurses in hospitals would help effectively identify the problems related to safety care activities and provide safer care. This study was only conducted on nurses of university hospitals, so subsequent studies should examine all types of healthcare organizations. Further studies are needed to test the scale in different contexts and cultures.

## Data Availability

The datasets during and / or analysed during the current study available from the corresponding author on reasonable request.

## Appendix

**Table 6:** Patient safety care activity scale for clinical nurses

Subcategory	Item
Security	1. Printouts containing medical information should be kept so that individuals cannot be identified.
	2. Log out when you are away.
	3. Do not use someone else’s ID and password when accessing the medical information system.
	4. The password used for work accounts should be changed periodically.
	5. Do not disclose patient information in private places.
	6. Do not look up medical information that is not related to work.
	7. Check and comply with institutional policies for medical information security.
Patient identification	8. Patients are identified using two indicators: patient name and registration number before medication, blood and blood product administration.
	9. When collecting specimens such as blood, the patient is identified using two indicators such as the patient name and registration number, and the name and registration number on the sample label match.
	10. Prior to treatment and procedure, patients are identified using two indicators: patient name and registration number.
	11. Ask open-ended questions when confirming patient names.
Operation (invasive procedure)	16. Before operation (procedure), check whether the area of surgery (procedure) is marked.
	17. Operation (procedure) patients perform a procedure to identify the correct patient, operation (procedure), and site for each stage of movement.
	18. When checking the area of operation (procedure), ask the patient orally to confirm it.
Medication	20. Always follow 5 Right when medication.
	21. Check the expiration date before medication.
	22. You Know the precautions when administering high-risk drugs (heparin, insulin, etc.) and how to deal with side effects.
	23. Label and store high-risk and high-caution drugs
	25. Check whether or not the medication was brought when hospitalized, and manage to prevent the patient from taking the medication on arbitrarily.
Blood transfusion	26. Before blood transfusion, check the blood type test and antibody screening test results.
	27. Ask patients about blood type and name in open-ended questions before blood transfusion
	28. Explain the purpose and method of blood transfusion and side effects of blood transfusion to the patient before blood transfusion.
	29. If side effects occur during blood transfusion, stop immediately and report to the doctor.
	30. Blood is transfused within 30 min of dispensing.
	31. Do not inject blood and intravenous injections at the same time.
Management of infection	32. Hand washing is performed before contacting the patient and before clean/sterile treatment.
	33. Hand washing is performed after contact with blood and body fluids.
	34. Hand washing is performed after contact with the patient and the patient’s surroundings.
	35. Infected patients are managed according to the guidelines for managing infected patients.
	36. Medical waste is collected in designated collection containers in accordance with bylaws.
	37. Separate and store contaminated laundry and other laundry according to the bylaws.
Management of falls and sores	38. Perform an initial patient assessment using a fall risk assessment tool.
	39. If there is a change in patient condition, medication, etc., re-evaluate the risk of falls.
	40. Share information about patients at high risk of falls.
	41. Provide fall prevention education to patients and caregivers at risk of falling.
	42. Perform appropriate fall prevention activities (to go to the toilet before bedtime, side rails, lighting, etc.) according to the risk of falling.
	43. Assess the risk of bedsores in all hospitalized patients using a bed sore assessment tool.
	44. Share information about people at high risk for bedsores with colleagues.
	45. Depending on the risk of bedsores, appropriate pressure sores prevention activities (change of position, use of support supplies, change of bedding, etc.) are carried out.
	46. Regularly observe the area where pressure sores can occur, and perform appropriate care (dressing, etc.) if necessary.
Management of firefighting	47. The hospital has a firefighting plan and you knows it.
	48. You Knows what to do in case of fire.
	49. You participate in fire drills regularly.
	50. You know where to evacuate in case of fire.

## Abbreviation

EFA: Exploratory Factor Analysis
